# A Misguiding Osteoid Osteoma in the Bicipital Tuberosity of the Radius

**DOI:** 10.1155/2016/6428137

**Published:** 2016-07-17

**Authors:** Konstantinos Ditsios, Nikolaos Papadakis, Ioannis Theodoroudis, Lazaros Kostretzis, Panagiotis Konstantinou, Iosafat Pinto, Anastasios Christodoulou

**Affiliations:** First Department in Orthopaedics and Trauma Surgery, Aristotle University of Thessaloniki, Georgios Papanikolaou Hospital, Exohi, 57010 Thessaloniki, Greece

## Abstract

Osteoid osteoma is a benign bone tumor that appears most frequently in young patients. It is more common in males and it concerns mostly the long bones of the lower limb. A 20-year-old young woman presented to our outpatient department with pain in her left elbow. The symptoms began four years ago. At first, her symptoms were attributed to ulnar neuritis, confirmed by nerve conduction studies. In the following two years, she had undergone two surgical operations for decompression of the ulnar nerve. As a result, she reported poor results, which forced her to take frequently anti-inflammatory drugs for some years. When the patient presented to us, we planned a three-phase bone scan and an elbow MRI, which revealed the lesion. Based on the image findings of osteoid osteoma, we proceeded to the surgical removal of the tumor. Since then, the patient is pain-free and has a full range of motion of the affected elbow. Osteoid osteoma usually mimics multiple pathologies in the upper limb especially joint disease posing a challenge for the physician. The diagnosis requires high index of suspicion and a prompt diagnostic and surgical management.

## 1. Inroduction

Osteoid osteoma is a relatively common benign bone tumor that appears most frequently in young patients. Its distinct feature is night pain that is relieved by anti-inflammatory drugs. It is more common in males and it concerns mostly the long bones of the lower limb (femur and tibia). Osteoid osteomas are very rare in the elbow region and even more in the bicipital tuberosity of the radius.

We therefore present a rare case of a 20-year-old woman with persisted pain in her elbow as a result of an osteoid osteoma that was falsely treated surgically as ulnar neuritis.

## 2. Case Report

A 20-year-old right-handed woman presented with pain on her left elbow radiating along the radial side of the forearm since the age of 14. At that time, our young patient had undergone surgical decompression of ulnar nerve at the cubital tunnel for the first time due to ulnar neuritis confirmed by nerve conduction studies. One year later, medial epicondylectomy was performed additionally. Because of poor results, nerve conduction studies were repeated and concluded that there was no ulnar nerve lesion.

The young patient presented at our department four years later and she was methodically examined to investigate her chronic pain. At first, a thorough history was taken. Apart from two surgical decompression procedures of the ulnar nerve, she reported no previous elbow trauma and no previous medical history. Physical examination was carried out and revealed full range of motion of the elbow. She had negative Tinel's sign and Phalen's test and no tenderness at the epicondyles.

**Figure 1:**
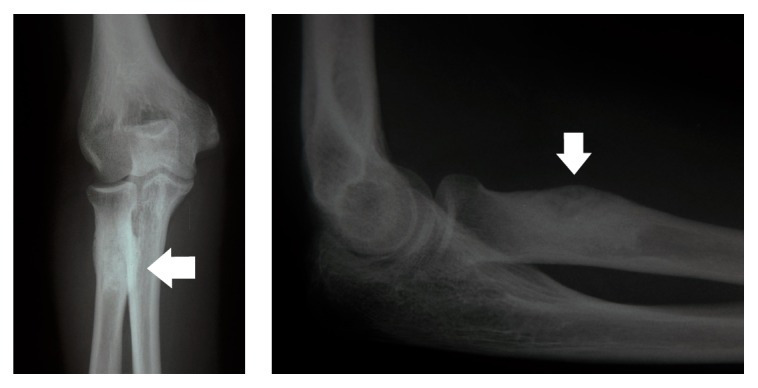
Radiograph images of the patient's elbow upon initial examination. Periostitis' signs and an image of “onion leaves” along with bone sclerosis at the bicipital tuberosity of the radius.

Since there were no significant clinical findings; a radiographic examination of her left elbow was undertaken. Radiographs showed an area of dense mineralization along with surrounding reactive sclerosis at the bicipital tuberosity of the radius (Figure 1).

**Figure 2:**
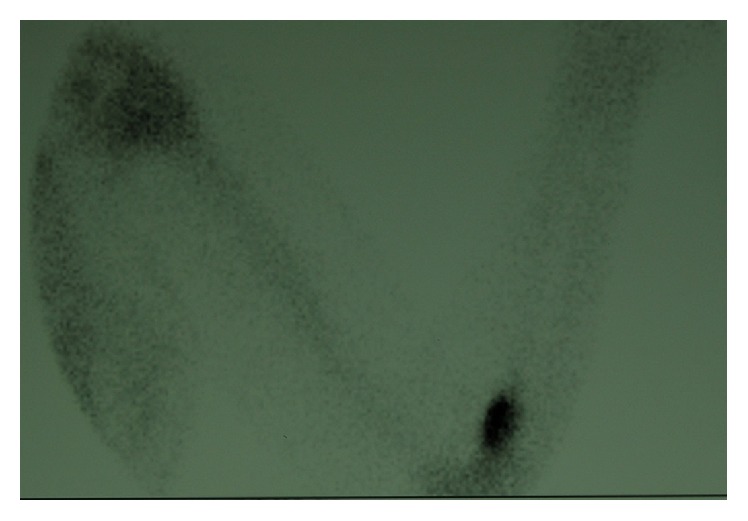
Bone scan image with Tc^99^ showing increased focal uptake at the bicipital groove.

To confirm the diagnosis, further imaging with a three-phase bone scan (Figure 2) and an MRI control of the region was made. The bone scan showed a high tracer uptake during all phases in the proximal end of the radius. The MRI scan revealed an intraosseous lesion that had no expansion at the surrounding tissues.

Surgical removal of the tumor was decided. The lesion was excised en bloc, under general anesthesia and tourniquet application. We used demineralized bone matrix graft to fill in the gap of the radius. There was no need to transpose the bicipital insertion in the radius.

Postoperatively, no evidence of elbow instability was observed. The diagnosis of the osteoid osteoma was confirmed histologically.

**Figure 3:**
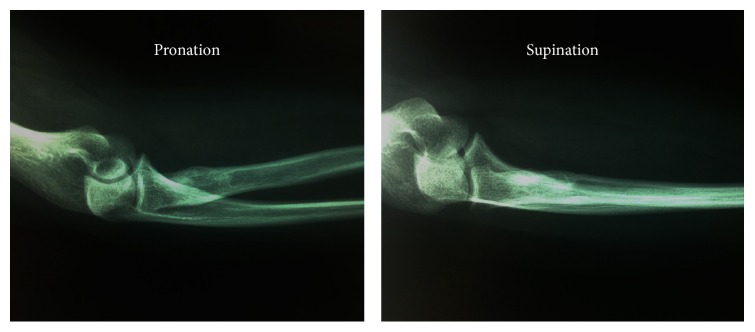
Radiograph images of the patient's elbow in pronation and supination, 8 years postoperatively with no signs of recurrence.

Since then, our patient has been pain-free and reports no need for painkillers. There was no need for physiotherapy to obtain full motion of the elbow. The normal follow-up visits for the patient were one after 6 weeks and a second after one year. Eight years after the operation, the patient visited again our outpatient department, had a full range of motion, and reported not consuming any painkillers for her elbow. She was fully satisfied by the operation and the final outcome (Figure 3).

## 3. Discussion

Osteoid osteoma is a benign bone tumor that occurs in the first three decades of life in more than 80% of the cases. It is more common in males than females and it represents about 12% of all benign bone tumors. It is more frequent in the long bones of the lower limbs (about 50% of the cases) and the spine represents the second most frequent site [1]. The pattern of pain is typical and is quite characteristic. It is persistent, gets more intense during the night, and subsides only with anti-inflammatory therapy or aspirin.

In our case, the tumor was in the upper extremity of the radius. The elbow is rarely a site of the tumor [2–4]. In some series, osteomas of the upper part of the radius consist 1% to 10% of all of the “elbow cases.” To our knowledge, only three cases of osteoid osteoma localized at the bicipital tuberosity of the radius [5, 6] have been reported.

The diversity of the symptoms and the lack of high suspicion result in a delay of the diagnosis. Especially in cases where the tumor lays close to a joint, the diagnosis is misled by pain due to arthritis and all the patients are treated for arthritic conditions. This delay in the diagnosis is supported by the literature and varies from 15 months to two years! Our patient's diagnosis was established after four years, which appears to be the longest in the literature.

X-rays are quite characteristic in cases of osteoid osteomas because of the nidus. However, in cases of small bones or when the lesion is located on the bone surface, signs inflammation of the periosteum are present without the characteristic nidus and diagnosis is harder. Our young patient represents such a case, whose X-rays revealed a reactive periostitis lesion that could be mistaken for any other kind of tumor. In order to set the right diagnosis apart from the X-rays, a CT scan and a three-phase bone scan can be very useful [7]. Even with these examinations, identification of the tumor is difficult. In our case, we preferred to perform an MRI of the region, in order to show any possible expansion of the tumor to the regional soft tissues.

The treatment consists of removal of the tumor along with the nidus. Only in very rare cases removal is not indicated when the patient is painless and there are no symptoms. Until some years ago, en bloc removal of the tumor was the only treatment available. During the last decades, however, CT-guided mini-incision surgery and CT-guided radiofrequency ablation seem to become more popular [8–10]. Even arthroscopical removal of the tumor has been reported [11, 12]. All methods seem to have satisfactory results [9, 10, 13]. Since we did not have the opportunity of radiofrequency ablation in our hospital, we performed open removal of the tumor with excellent results and two years after the operation there is no recurrence of the tumor.

The diagnosis of osteoid osteoma can be difficult. Complicated anatomy and patients' tendency to correlate their symptoms with trauma or exhaustion often misguide clinicians and lead to ineffective treatments. Doctors should have high suspicion in cases of chronic bony pain and diagnosis can be reached following thorough physical and imaging examination.
